# Stuck in space but not in time: multiple-scale resource selection in a stationary prey

**DOI:** 10.1186/s40462-026-00631-3

**Published:** 2026-02-11

**Authors:** Roel May, Craig Ryan Jackson, Justin Wanda, Robert Fyumagwa, Kjetil Bevanger, Eivin Røskaft

**Affiliations:** 1 Norwegian Institute for Nature Research (NINA), Høgskoleringen 9, Trondheim, NO-7034 Norway; 2 Tanzania Wildlife Research Institute (TAWIRI), Serengeti Wildlife Research Centre (SWRC), P. O. Box 661, Arusha, Tanzania; 3 Department of Biology, Norwegian University of Science and Technology (NTNU), Realfagbygget, Trondheim, NO-7491 Norway

**Keywords:** Human disturbance, Predation risk, Impala, Moon phase, Landscape, Habitat

## Abstract

**Background:**

Prey species need to adjust their habitat selection either temporally or spatially to reduce predation risk or human disturbance. We tested the risk allocation hypothesis that poses that foraging is higher in low-risk habitats and during low-risk periods for impala (*Aepyceros melampus*) in the Serengeti Ecosystem. We expected impalas to select ranges that balance long-term predation risk and forage availability and adjust their habitat utilization within their ranges temporally to balance short-term predation risk and energy intake.

**Methods:**

We modelled multi-scale resource selection for 36 GPS-tracked impala (2016–2018) to disentangle spatial and temporal trade-offs between forage acquisition and predation risk using resource selection functions at the landscape and within-home-range level. We contrasted resource selection inside and outside of Serengeti NP (SNP) for woody cover, forage availability (vegetation heterogeneity, NDVI) and risky places (proximity to water, terrain ruggedness). We also modelled responses to diel and lunar cycles in space use.

**Results:**

Impalas attuned their resource selection both towards maximizing forage availability and minimizing risk exposure, especially at the landscape level. While home-ranges were generally placed in more homogeneous woodland close to water sources, impalas preferred to use more heterogeneous, open and rugged patches within their home-ranges. They adjusted their responses outside SNP by placing their home-ranges in less rugged terrain and farther from water sources. Impalas balanced foraging and risk temporally by adjusting their preferences to circalunar and diel patterns.

**Conclusions:**

Impala attuned their home-range placement and utilization according to the ‘landscape of fear’ hypothesis inhabiting a ‘squeezed’ landscape. Here, prey to a functionally diverse carnivore guild clearly balanced their space use between food acquisition and spatio-temporal risk avoidance. Humans, however, acted as a ‘super-predator’ eliciting a stronger behavioural response compared to natural predators. Following the risk allocation hypothesis, the presence of humans was of such an intensity and predictability that it changed the anti-predator responses in impala compared to the adjacent natural system with carnivores only. A functional response in habitat selection indicated that impala avoided long-term spatial risk in home-range placement, while within their home-ranges short-term temporal risk mimicked the diel patterns observed within the protected area with natural predators only.

## Background

Anthropogenic disturbances now significantly impact nearly every habitat on Earth, and human-induced environmental changes are impacting most species and ecosystems, either directly or indirectly [1]. These processes have contributed significantly to the degradation of African savanna ecosystems, particularly in areas with high agricultural and pastoral potential [2]. Natural habitats are cleared for agricultural production and human settlement, while growing livestock numbers increase resource competition with wild ungulate populations and result in habitat modifications [3] as well as declining ungulate populations [4]. Conflicts related to depredation of livestock have also led to declines in large carnivores in areas adjacent to protected areas [5–7]. While reduced carnivore presence as a result of anthropogenic activities in human-altered landscapes may provide ungulate prey species with a spatial refuge [8, 9], it may also lead to an ecological trap through as these areas are frequently characterised by increased risk of mortality from poaching [10, 11]. Ungulates can mediate predation and disturbance risks by adjusting their habitat choice to accommodate spatio-temporal risk patterns [12]. The field of movement ecology [13] has developed rapidly concurrent to technological improvements in capturing data [14]. It has taught us much on the environmental factors affecting movement decisions [15], and how animals may be able to track resources [16]. This has led to novel concepts on how ungulates optimize food acquisition in ‘energy landscapes’ [17] and balance this against avoiding being preyed upon in ‘landscapes of fear’ [18]; not in the least in the face of anthropogenic landscape change [19, 20].

Although protected areas aim to safeguard wildlife populations from habitat loss, degradation and detrimental human activities, even in strictly protected areas wildlife populations can decline. Populations of large mammals, mainly herbivores, have declined with an average of 59% in the past decades in protected areas across the African continent [21]. Landscape degradation caused by human activities along the edges of protected areas, including adjoining partially protected areas, may not only impact wildlife populations outside the boundaries of strictly protected areas, but within the principal protected area too [22]. Due to the far reaching (spatial) effects of anthropogenic activities, wildlife populations within well protected areas may not necessarily escape these detrimental processes. Disturbance caused by human activities can alter animals’ perception of humans as potential predators; the response to disturbance can in this context be studied in the same way as the response to predation risk [23, 24].

Animals that are preyed upon need to balance the acquisition of food with avoiding the risk of predation [25, 26]. As a response to predation risk, animals have been shown to shift their habitat use in space and time, as well as increase vigilance and group size [27–29]. In order to reduce predation pressure, prey species need to adjust their ranging behaviour and habitat selection to minimize spatial overlap with and proximity to predators [30]. The extent to which a species is able to balance foraging and cover from predation will also depend on habitat availability and exemplifies a functional response in resource selection [31]. Prey species can respond proactively to predation risk as long as related cues vary over time and across space [32] and are predictable [33]. Negotiating the trade-off between food and safety may be balanced either by responding to temporal variation in risk (the risky times hypothesis) or to spatial variation in risk (the risky places or ‘landscape of fear’ hypothesis) [18, 34–36]. The risk allocation hypothesis poses that foraging is higher in low-risk habitats and during low-risk periods [26]. The strength of anti-predator responses also depends on the duration, intensity and predictability of predation risk [28, 37, 38]. A recent empirical study in Africa found that responses of ungulates to long-term spatial predation risk interacted with short-term temporal risk, such that vigilance levels increased in risky places only during risky times [39]. Since anti-predator responses may occur at varying spatiotemporal scales, these should be investigated at multiple spatial and temporal scales; including habitat use, movement patterns, seasonality and activity cycles [32].

The impala (*Aepyceros melampus*) is a medium-sized antelope inhabiting bushland, woodland, and savanna habitat in eastern and southern Africa [40]. Impalas are prey to a functionally diverse carnivore guild, including lions (*Panthera leo*), leopards (*Panthera pardus*), spotted hyena (*Crocuta crocuta*), cheetah (*Acinonyx jubatus*) and African wild dogs (*Lycaon pictus*) [41]. As impala are affected in their behaviour in space and time by the presence of both carnivores and human disturbance [42], they form a good model species to assess effects of spatiotemporal patterns in risk allocation. The impala is a non-migratory and gregarious species that inhabits small home-ranges, and as a mixed feeder shift their dietary preferences between grazing and browsing seasonally [43]. Also, anti-predator responses have been shown to be stronger for smaller browser/mixed-feeder ungulates such as the impala [29]. Their plasticity in ranging behaviour and resource selection within their environment therefore must be adjusted to local predation risk and human disturbance. We expected that impalas seek to utilize (1) areas of general suitability balancing long-term predation risk and forage availability at the landscape level, and (2) adjust their habitat utilization within their home-ranges temporally to balance short-term predation risk and energy intake. More specifically, we hypothesize that impalas prefer to utilize heterogeneous areas with good forage quality (NDVI), availability of surface water [44], woody cover for feeding, resting and hiding habitat, and simultaneously avoid potentially risky places, i.e. proximity to water preferred by lions [45], rugged terrain preferred by African wild dogs [46] and woody cover for leopards [47]. These factors may vary across spatial scales as well as temporarily due to differential effects of forage competition with livestock and poaching pressure outside protected areas that often supersede the relative risk posed by carnivores (especially when anthropogenic effects result in lower carnivore densities than inside protected areas), versus predation risk by a diverse carnivore guild inside protected areas. Impalas have in addition also been shown to adjust their space use according to diel and lunar cycles to reduce predation and disturbance [48, 49]. Following the recommendation by [32], we modelled multi-scale resource selection in impala to disentangle spatial and temporal trade-offs between forage acquisition and predation risk whilst also controlling for the potential effects of lunar phase [50]. By conducting this analysis for impalas occurring inside and outside a protected national park, we could assess the relative effects of predation (inside) and that of (reduced) predation and human risk (outside) on impala habitat selection.

## Methods

### Study area

**Fig. 1:**
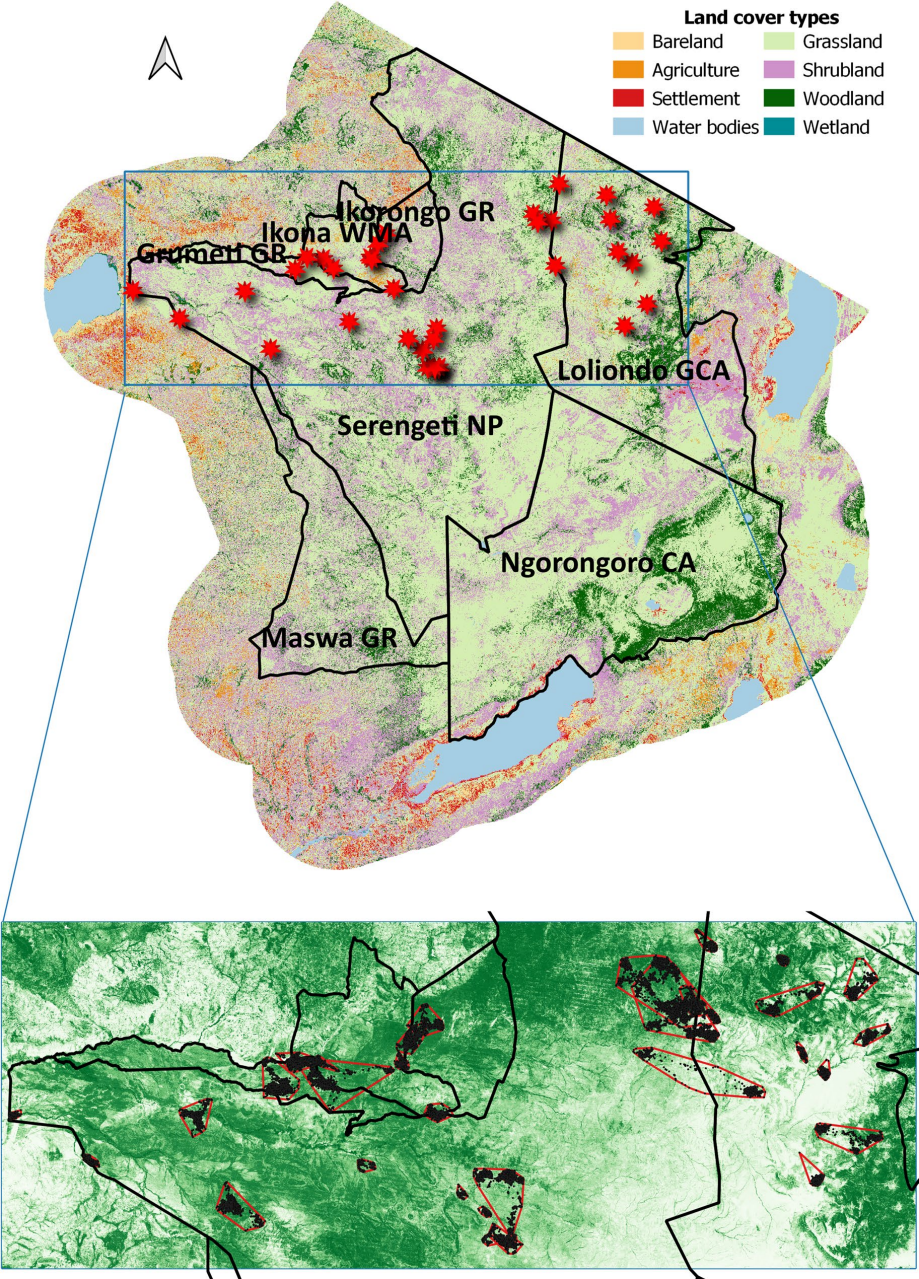
Locations of tracked impala (red stars) within the Serengeti Ecosystem (top panel), and minimum convex polygons and relocations shown on top of woody cover (lower panel)

The Serengeti Ecosystem (ca. 25,000 km^2^) in Tanzania is centred around the Serengeti National Park (SNP; 14,763 km^2^); a world-renowned biodiversity hotspot, a UNESCO World Heritage Site, and Man and Biosphere Reserve [51]. SNP is a stronghold for large populations of ungulates [52] and a varied carnivore assemblage [53, 54]. Impala are a common resident species in the Serengeti Ecosystem, with an estimated abundance of ca. 75,000 (± 9,000) individuals [52]. This core area is surrounded by partially protected areas with different forms of land management, including Ngorogoro Conservation Area (NCA), another UNESCO Mixed World Heritage Site and Biosphere Reserve; Loliondo Game Controlled Area (LGCA); IKONA Wildlife Management Area (IWMA); Maswa Game Reserve (MGR); Grumeti Game Reserve (GGR) and Ikorongo Game Reserve (IGR). Outside of these buffer areas, the remainder of the study area (outermost areas without borders in Fig. 1) is unprotected land with high levels of human presence and agricultural activity. SNP is strictly protected, and only photographic safari-based tourism is allowed. While pastoralism, settlements and resource extraction are allowed in NCA and LGCA, in IWMA and the Game Reserves both consumptive and non-consumptive tourism are permitted and regulated [55]. Even though both core and buffer areas are protected, illegal activities are still pervasive and include illegal bushmeat hunting, timber extraction and livestock grazing [22, 56]. Both in LGCA and IWMA, where humans live side-by-side with wildlife, conflicts occur with respect to livestock depredation and retaliation [57, 58], livestock competition [59–61], and poaching [62, 63]. However, while carnivores were thriving in the western Game Reserves, e.g [64], the presence of carnivores have declined drastically in LGCA [65]. This decline, combined with declines in other prey species and prevalent poaching, increases risk exposure for impalas [42, 65]. Consequently, risks to ungulates in SNP are dominantly characterised by natural predation. In contrast, the adjoining management areas present a host of anthropogenic risks in addition to predation risk [42], despite the latter often being lower due to declines in carnivore densities in the mixed land-use areas [5]. We set the spatial extent of the study to include the landscape surrounding the area utilized by the monitored impala (Fig. 1; bounding box: 595000, 9725000, 795000, 9800000; EPSG:32736 – WGS 84 UTM zone 36 S).

### Collaring and tracking

In total 36 adult female impalas were tracked with satellite Global Positioning System (GPS) collars during the period 27th of April 2016 to 30th of September 2018 across the Serengeti Ecosystem (including SNP, GGR, IGR, IWMA, LGCA). TAWIRI veterinary and research staff members conducted all immobilizations and oversaw the fitting of GPS-collars (1D Iridium satellite GPS collars with accelerometer, Lotek Wireless Inc., Canada) to all impalas. Mostly, we immobilized impalas by darting them (*N* = 30) using 1 cc darts (Pneu-Darts, Incl.) shot from a Model 196 Pneu Dart cartridge-fired projector at a reachable distance of about 10–15 m targeting the shoulders or thighs. The darts had a mixture of either 3–4 mg etorphine hydrochloride (M99) and 10-15 mg xylazine hydrochloride; or 2–3 mg etorphine hydrochloride, 3 mg medetomidine hydrochloride and 18–20 mg azaperone tartrate. Alternatively, we also captured impalas using a net and physically restrained them (*N* = 6). All tracked animals responded well to the reversals and we observed no negative post-handling effects. In total 211,767 hourly relocations were registered on their movements and used to investigate resource selection. The individuals were tracked for on average 248 days ± 178 SD (range: 17–568, median: 221 days), rendering on average 5882 ± 4287 SD (range: 415–13,622, median: 4426) relocations per individual. The 100% Minimum Convex Polygons (MCP), indicating their area of utilization, of these individuals was on average 37.2 km^2^ ± 52.3 SD (range: 3.5–202.2; median: 16.3 km^2^). Mean hourly step length was 125 m ± 170 SD (range: 0–7654; median: 69 m).

### Spatial and temporal covariates

We modelled resource selection within the study area using the following covariates. We included area heterogeneity using a Shannon diversity map derived from the land cover map [66] within a 1 km circular buffer using the focalWeight function of the raster package [67]. We derived terrain ruggedness from the digital elevation model obtained from NASA Shuttle Radar Topography Mission (SRTM) [68]. We obtained woody cover from the Global Forest Cover Change (GFCC) dataset for 2015 [69]. We calculated Normalized Difference Vegetation Index (NDVI) from SENTINEL-2 images for each season in 2016–2018 obtained from the SentinelHub. For this, within each season we chose those acquisition dates with highest quality (i.e. least cloud cover) closest to mid-season. We calculated proximity to river from the Serengeti GIS & Data dataset on rivers and small drainages from the Greater Serengeti-Mara Ecosystem​ digitized from 1:50,000 map sheets [70]. Finally, we resampled all spatial covariates to a resolution of 30 m. We defined seasons as short dry (Jan-Mar), long rain (Apr-Jun), long dry (Jul-Sep) and short rain (Oct-Dec). We downloaded percent moon illumination from timeanddate.com for SNP (download date: 06.02.2019).

### Resource selection analyses

To evaluate selection and avoidance of environmental features in the impalas’ habitat utilization, we employed a use–availability framework comparing environmental covariates of locations which were known to have been used to (random) locations assumed to have been available for selection using resource selection functions [71]. We investigated resource selection at the landscape level and home-range level (2nd and 3rd order selection, respectively) [72], by constructing resource selection functions (RSF) using generalized linear mixed-effects models with a binomial distribution using the glmer function of the lme4 package [73]. The RSF assessed the influence of spatial covariates (percent woody cover (WC), terrain ruggedness index (TRI), Shannon vegetation diversity (SVD), proximity to rivers (ProxW) and NDVI) both inside and outside of the SNP (InOut interaction term) and/or for lunar phase (Moon interaction term). The models controlled for the random effects of management area (Fig. 1), individual and at the home-range level also season nested with year.

We contrasted hypothesis-based RSF models (Table 1) at the landscape and home-range level using an Information Theoretic approach [74] using Akaike’s Information Criterion (AIC). The Information Theoretic approach is preferred in multi-hypothesis testing, whereby the most parsimonious model – and therefore the most supported hypothesis – corresponds to the model with the lowest AIC score. Besides one generic habitat model (woody cover only) used as a null model, the models captured different risk-coping strategies. Generally (model set *G*), impala resource selection can influence their fitness and survival by either maximizing forage availability (*f* models), minimizing exposure to predation risk (*r* models), or striking a balance between these two (*fr* models). Impalas may be able to further attune their area use spatially according to the risky spaces hypothesis (i.e. differential response to humans versus natural predators inside versus outside SNP where carnivores have declined; model set *S*), temporarily according to the risky times hypothesis (i.e. different responses due to lunar luminance; model set *T*) or spatio-temporally according to the risk allocation hypothesis (i.e. combination of the previous two; model set *ST*). While spatially explicit models could be evaluated for both the landscape and home-range levels, temporally explicit and spatio-temporal trade-off models could only be constructed for the home-range level. We compared relative support for different model sets by calculating the ratio between the overall mean ΔAIC across all models to the mean ΔAIC for the given model set.

**Table 1:** Resource selection function model structures and associated predictions

	Nr	Prediction	Scale		Model structure
	*0*	Generic resource selection	Landscape resource selection	Home-range resource selection	WC
Risky	*Gf*	Maximization of forage availability			WC + SVD + NDVI
	*Gr*	Minimization of predation risk exposure			WC + ProxW + TRI
	*Gfr*	Trade-off between forage availability and predation risk			WC + SVD + NDVI + ProxW + TRI
Risky places	*Sf*	Spatially adjusted maximization of forage availability			(WC + SVD + NDVI) * InOut
	*Sr*	Spatially adjusted minimization of predation risk exposure			(WC + ProxW + TRI) * InOut
	*Sfr*	Spatially adjusted trade-off between forage availability and risk exposure			(WC + SVD + NDVI + ProxW + TRI) * InOut
Risky times	*Tf*	Temporally adjusted maximization of forage availability			(WC + SVD + NDVI) * Moon
	*Tr*	Temporally adjusted minimization of predation risk exposure			(WC + ProxW + TRI) * Moon
	*Tfr*	Temporally adjusted trade-off between forage availability and risk exposure			(WC + SVD + NDVI + ProxW + TRI) * Moon
Risky trade-off	*STf*	Spatio-temporally adjusted maximization of forage availability			(WC + SVD + NDVI) * (InOut + Moon)
	*STr*	Spatio-temporally adjusted minimization of predation risk exposure			(WC + ProxW + TRI) * (InOut + Moon)
	*STfr*	Spatio-temporally adjusted trade-off between forage availability and risk exposure			(WC + SVD + NDVI + ProxW + TRI) * (InOut + Moon)

All models in addition controlled for the random effects of management area, individual and at the home-range level also season nested with year. The models represent different generic (*G*), Spatial (*S*), Temporal (*T*) and spatio-temporal (*ST*) responses to predation risk (*r*), forage availability (*f*), or both (*fr*). The first seven models were used for the landscape level, while all were used for the home-range level. The contrasted models include the explanatory Spatial covariates Woody cover (WC), Shannon vegetation diversity (SVD), terrain ruggedness (TRI), proximity to water (ProxW) and forage productivity (NDVI) and locations with regard to the SNP boundaries (InOut) and/or lunar cycle (Moon)

To render an approximate 1:10 use-to-available ratio for all our models, we randomly placed 10 times the average number of relocations for any given hour (i.e. 10 × 8823.6 = 88,236) across the study region as well as within the 100% MCPs for, respectively, the landscape level and home-range level analyses. At the landscape level, 260 points had to be discarded due to missing background data. At the home-range level, the number of random points placed within the MCPs was proportional to the number of relocations per individual to ensure a balanced design. In the landscape level analysis, we compared all random points across the study region (available) with a random subset of 10% of the random MCP points (used). We based the home-range level analysis on all MCP random points (available) and compared these to a random subset of 1/24th of all relocations (used). Thereafter, we assessed diel patterns in home-range level resource selection by running 24 separate model sets for each hour of the day based on the most parsimonious home-range model. Here, we compared all MCP random points (available) to all relocations for a given hour (used). This split modelling approach allowed us to assess diel patterns for each covariate separately and simultaneously avoid both temporal autocorrelation and potential overfitting issues.

We evaluated the predictive success of the most parsimonious landscape and home-range level models using k-fold cross-validation employing an adjusted kxv_lmer function [75] to accommodate glmer models (kxv_glmer). We trained the model on 90% of the data and testing the goodness-of-fit of the remaining 10% iteratively (we randomly binned the data into 10 classes) using Spearman-rank correlations, which were calculated between ten resource selection function bin ranks and area-adjusted frequencies for ten ‘test-training’ sets. We averaged the goodness-of-fit statistics for the 24 hourly home-range level models across these hourly models. We tested diel patterns in home-range resource selection by regressing 24 hourly model coefficients against a single raised-cosine (day-night activity) or double raised-cosine (dawn-dusk activity) daily pattern.

## Results

**Fig. 2:**
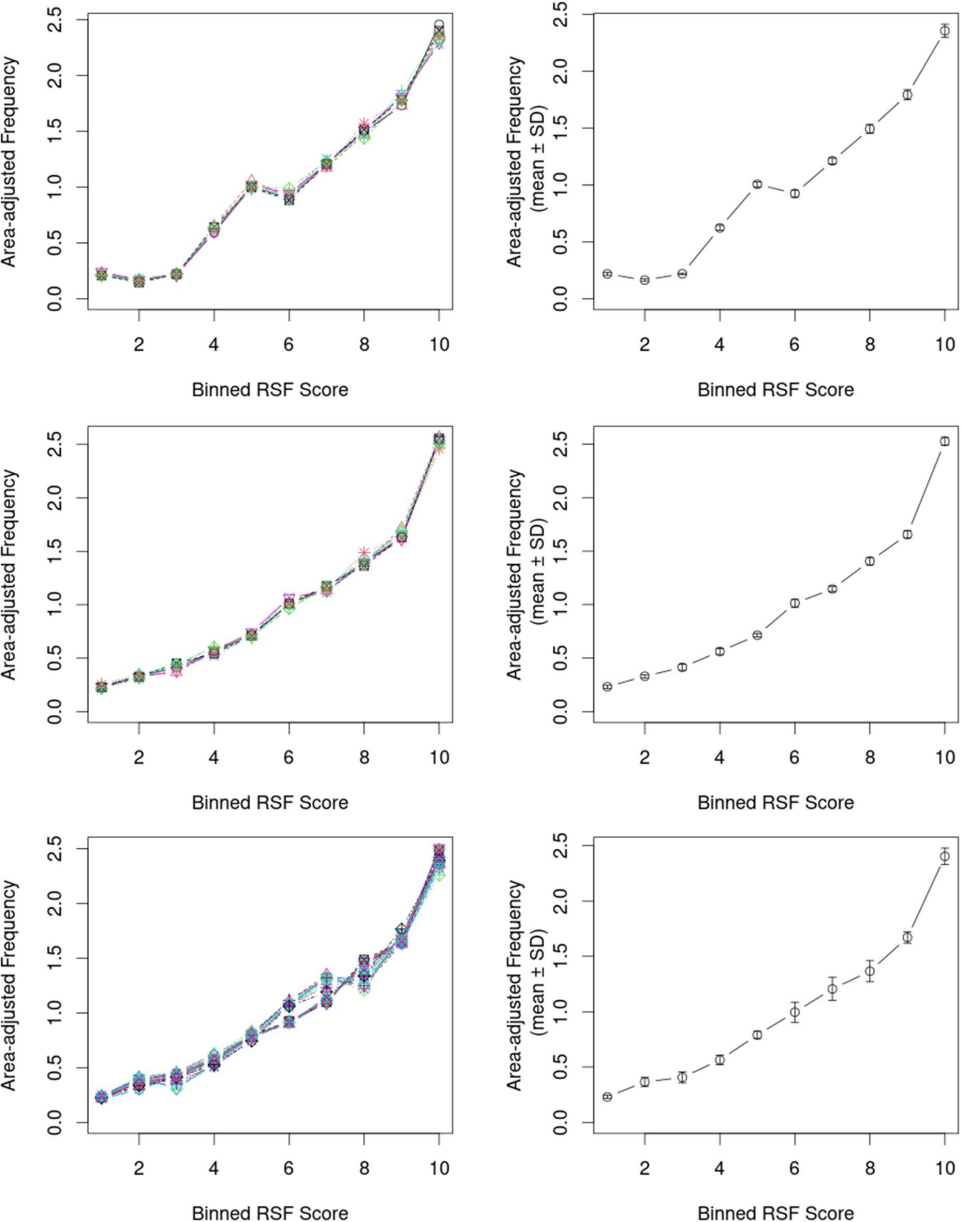
Cross-validation for landscape level (top) and home-range level (middle) and hourly (bottom) resource selection in impala within the Serengeti Ecosystem. Left-hand panels show the area-adjusted frequencies across bins for the different iterations. Right-hand panels show the averaged values including SD. Analyses were based on the most parsimonious model (*Sfr*)

Overall, our modelling showed that impalas attuned their resource selection both towards maximizing forage availability and minimizing risk exposure (Table 2). They adjusted these responses according to the human disturbance they were exposed to outside versus inside of SNP. Risk aversion was stronger at the landscape level, while they favoured forage availability stronger within their home-ranges. Of the 13 hypothesis-driven models, the *‘Risky places’ Spatially adjusted trade-off between forage availability and risk exposure* model (*Sfr*) had the highest parsimony both for the landscape and home-range level as indicated by AIC (Table 2). However, at the home-range level the *‘Risky trade-off’ Spatio-temporally adjusted trade-off between forage availability and risk exposure* model (*STfr*) was nearly as good as the home-range level *‘Risky places’* (*Sfr*) model with ΔAIC = 5 (Table 2). Generally, there was more support for models (based on comparing ΔAIC-ratios) including spatially differentiated responses (landscape level *S* models: ΔAIC-ratio of 1.45; home-range level *S* and *ST* models: ΔAIC-ratios of 1.39 and 1.38 respectively) compared to generic (*G*) or temporal (*T*) models (landscape level: 0.76; home-range level: 0.78 and 0.78). Impala responded more to forage availability and risk exposure jointly (*fr* models; ΔAIC-ratios of 2.25 and 3.06 for the landscape and home-range level respectively) compared to the other sets of models. While they responded more to risk exposure at the landscape level (*f* models: 0.53; *r* models: 1.53), at the home-range level impalas selected more strongly for forage availability (*f* models: 1.35; *r* models: 0.52). The predictive success of the most parsimonious (*Sfr*) landscape level RSF model showed a good fit (*r* = 0.976 [range: 0.952–0.976], *P* < 0.001), as did the home-range level RSF model (*r* = 1.000 [range: 1.000–1.000], *P* < 0.001). These are graphically shown in Fig. 2.

**Table 2:** Parsimony of regression models for landscape and home-range level resource selection in Impala within the Serengeti Ecosystem. The models are described in Table 1

Model	Landscape level			Home-range level		
	df	AIC	ΔAIC	df	AIC	ΔAIC
*0*	4	53 802	649	6	56 092	1 380
*Gf*	6	53 742	589	8	55 211	498
*Gr*	6	53 499	346	8	55 839	1 127
*Gfr*	8	53 427	274	10	55 041	329
*Sf*	10	53 734	582	12	54 968	256
*Sr*	10	53 211	58	12	55 554	841
*Sfr*	14	53 152	0	16	54 712	0
*Tf*				12	55 211	499
*Tr*				12	55 845	1 132
*Tfr*				16	55 046	333
*STf*				16	54 969	256
*STr*				16	55 559	847
*STfr*				22	54 717	5

**Fig. 3:**
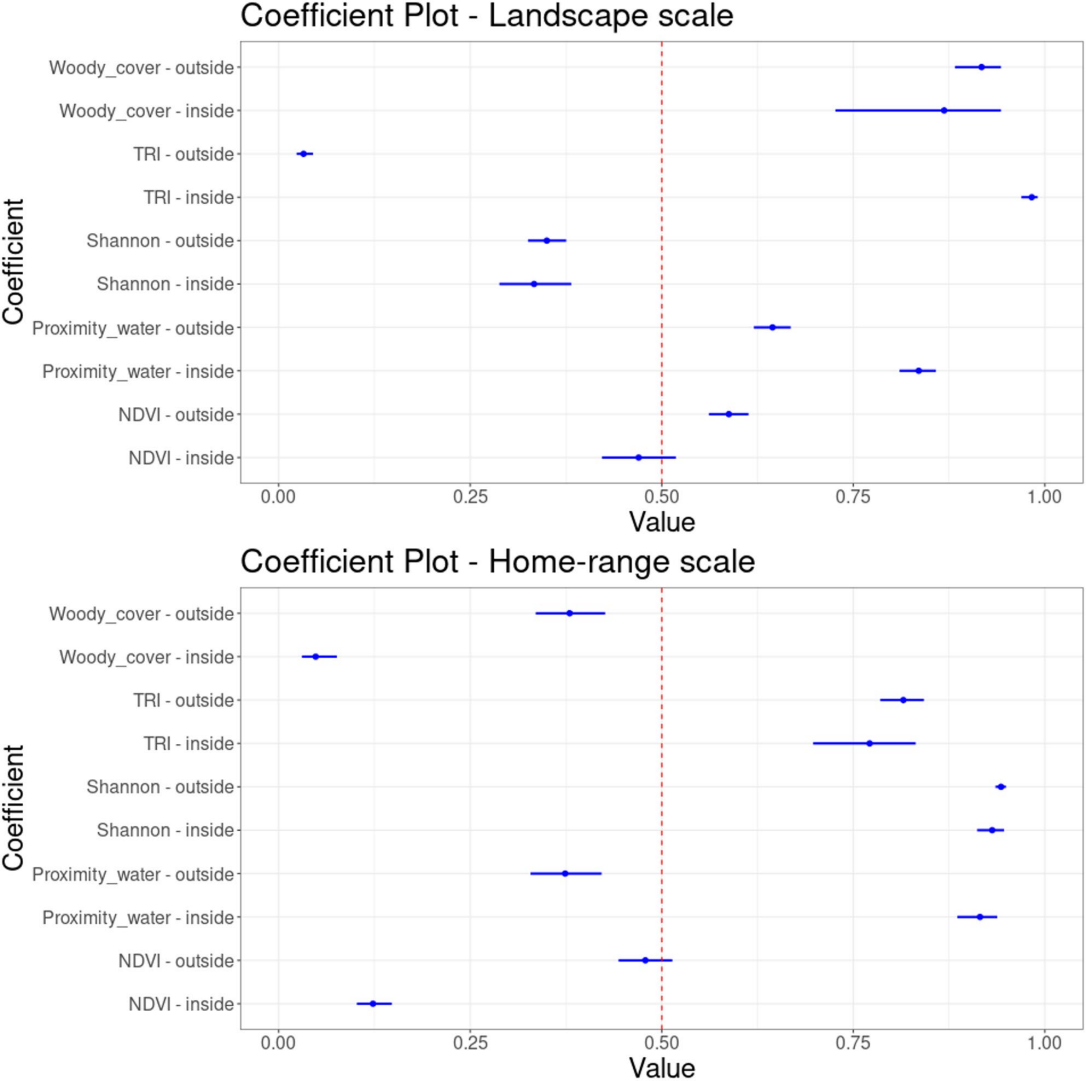
Relative probability of selection for resources inside and outside SNP at the landscape level (top panel) and home-range level (bottom panel) for the most parsimonious model (*Sfr*). Horizontal blue lines indicate within-model variance. Values above and below the dashed red line indicate, respectively, selection for and avoidance of the covariates

While home-ranges were generally placed in more homogeneous woodland close to water sources, impalas preferred to use more heterogeneous, open and rugged patches within their home-ranges (Fig. 3). Outside SNP, they placed their home-ranges preferably in less rugged terrain and farther away from water sources relative to their preferences inside SNP. This suggests a functional response in resource selection regarding their response to risk exposure which was lacking inside SNP. To test this, we calculated the correlation between individual landscape and home-range level selection ratios and found a functional response for proximity to water outside of SNP (Table 3).

**Table 3:** Individual functional responses in resource selection as measured by the pearson’s product-moment correlation estimates between landscape level and home-range level selection ratios (mean used / mean available)

Covariate	Estimate	*P* value
TRI - outside	-0.213	0.329
Shannon - outside	-0.292	0.176
Woody cover - outside	-0.315	0.144
NDVI - outside	0.081	0.713
Proximity water - outside	-0.480	0.020
TRI - inside	0.322	0.166
Shannon - inside	-0.263	0.262
Woody cover - inside	-0.129	0.587
NDVI - inside	-0.280	0.231
Proximity water - inside	-0.209	0.376

**Fig. 4:**
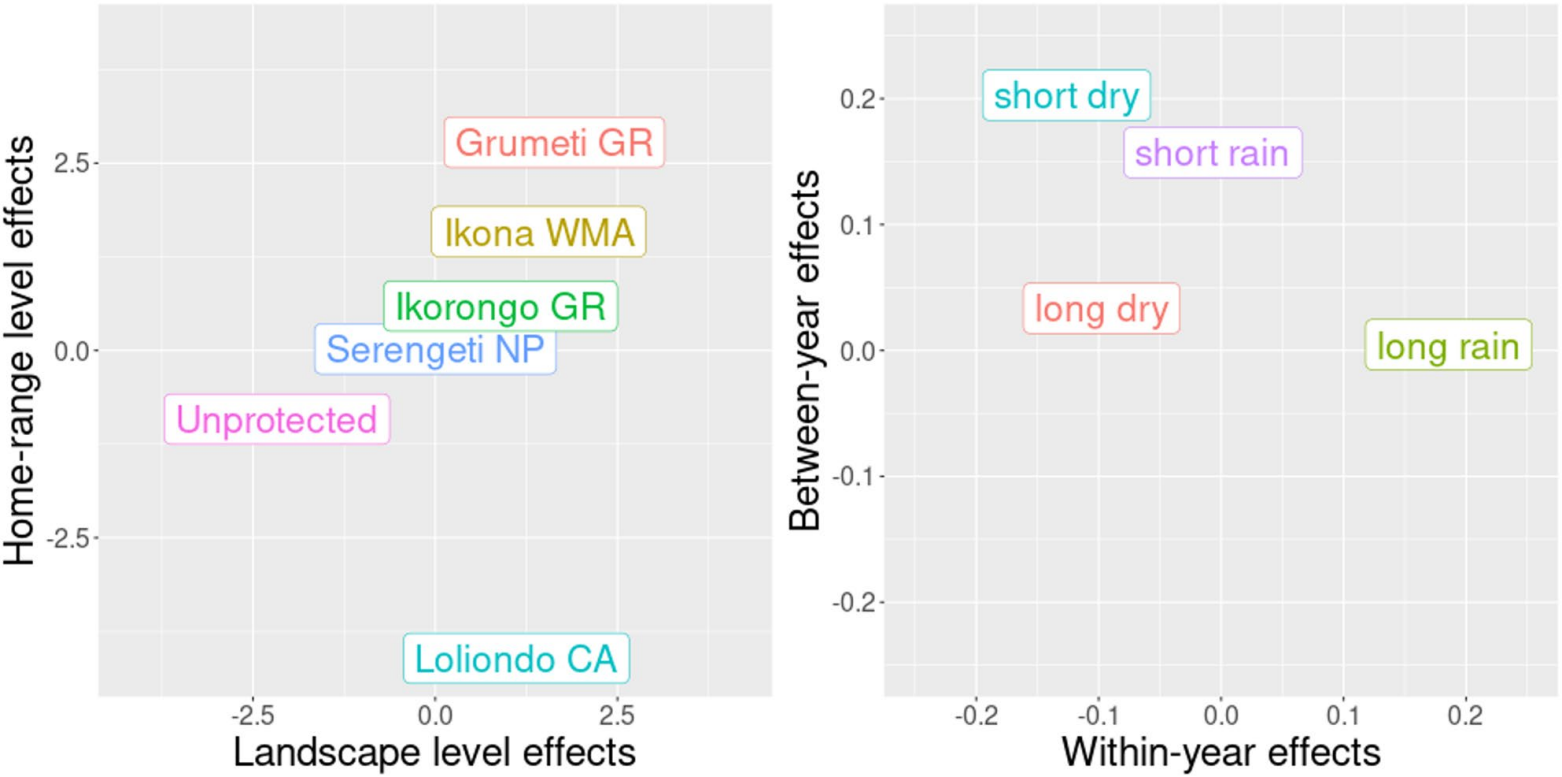
Conditional modes (i.e. random intercepts) at the landscape and home-range level for responses to different management areas and surrounding unprotected land in the Serengeti Ecosystem (left-hand panel), and for responses to within- and between-year variance within different seasons (right-hand panel). Conditional modes are given relative to the overall mean resource selection and based on the most parsimonious model (*Sfr*)

Further investigation of the random effects included in the most parsimonious model showed a clear effect of management areas on habitat selection (Fig. 4). At the landscape level, impalas had –not surprisingly– a lower habitat selectivity (i.e. lower random intercept) in unprotected land; while we found no such effect at the home-range level as they did not spend any significant amount of time in these areas. Relative to SNP (reference category), impalas had a higher habitat selectivity in the western buffer areas IWMA, GGR and IGR. In LGCA they showed a distinct lower selectivity within their home-ranges. Within their home-ranges, within-year seasonal effects were larger compared to between-year variation in seasonal effects (Fig. 4). This result was not unexpected as the impalas were only tracked within the span of three years. Impalas had a stronger habitat selectivity during the long rain season and less so during the short dry season, which also varied more between years.

**Fig. 5:**
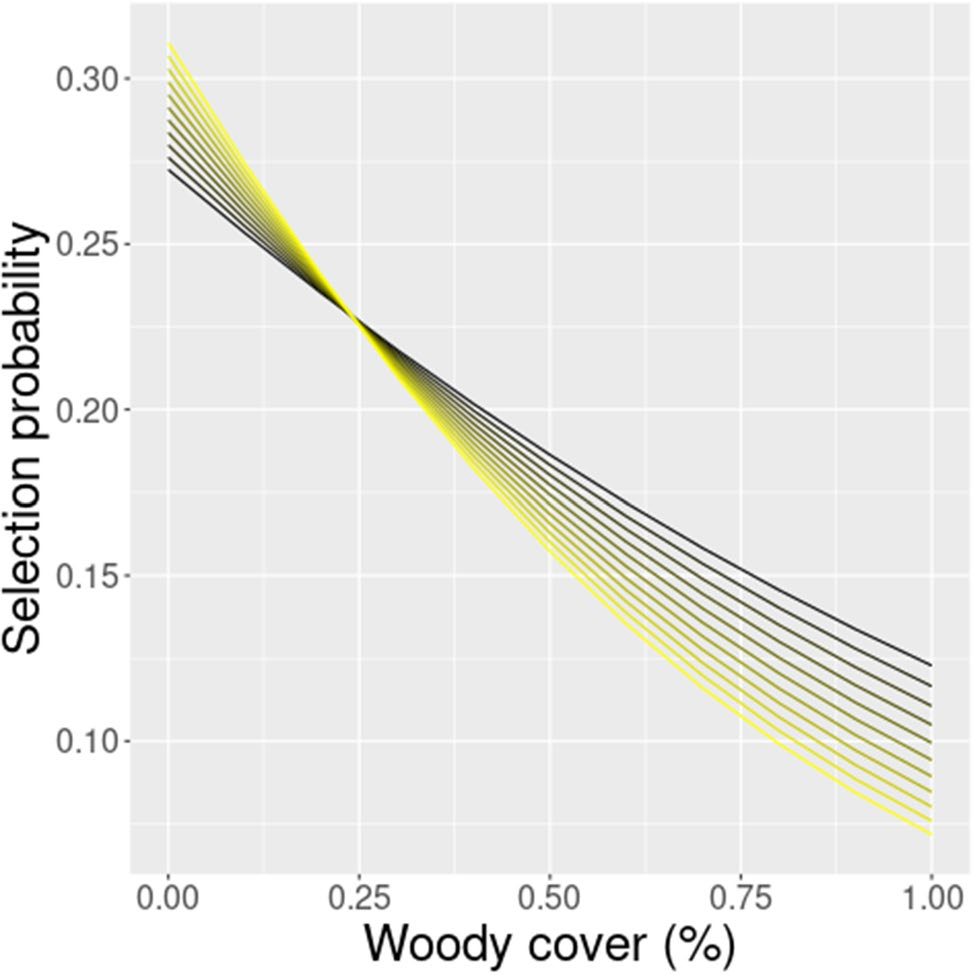
Change in selection for woody cover within home-ranges for different levels of lunar luminance in steps of 10% (0% – 100%; black to yellow); based on the ‘Risky trade-off’ model (*STfr*)

**Fig. 6:**
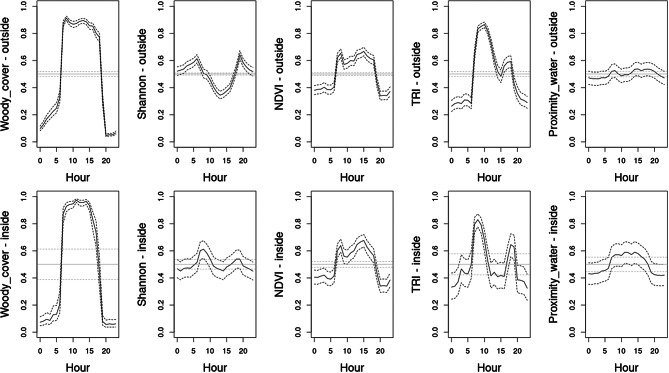
Diel patterns in within-home-range resource selection in impala in the Serengeti Ecosystem based on the ‘Risky trade-off’ model (*STfr*). Y-axis indicates the difference in selection probability (± hourly within-model variance) relative to the daily mean effect for each environmental covariate (mean set at 0 indicated by solid grey line and 95% confidence interval for mean within-model variance indicated with grey dotted lines)

Even though the spatially explicit *‘Risky places’* model (*Sfr*) had the highest parsimony, the next best model (*STfr*) indicates that impala resource selection may also be affected temporally. This *‘Risky trade-off’* model included a significant interaction term for woody cover responding to temporal changes in lunar luminance (F = 2.25; estimate: -0.078 ± 0.380, z = 2.048, *P* = 0.041; Fig. 5). During full moon nights, impalas selected for more open woodland compared to darker nights when they preferred more closed woodland. We reran the most parsimonious home-range level model also for each hour of the day to further investigate diel patterns in resource selection. The average AIC across the hourly home-range level RSF models was quite stable: 54,325.69 ± 246.60 SD, and showed a good fit across hourly bins (*r* = 0.994 [range: 0.976-1.000], *P* < 0.001). All covariates, both inside and outside SNP, showed a significant day-night pattern in resource selection but only a subset of covariates had clear dawn-dusk patterns (Table 4). Woody cover had by far the strongest day-night pattern, whereas proximity to water was less pronounced; both showed stronger avoidance during the night (Fig. 6). Dawn-dusk responses were negligible for both woody cover and proximity to water. Impalas preferred more heterogeneous vegetation during dawn-dusk compared to midday. Inside SNP, impalas had a higher preference for areas with higher NDVI and especially more rugged terrain during dawn-dusk. Both covariates had clear day-night patterns with selection of higher NDVI and ruggedness during the day.

**Table 4:** Diel patterns in within-home-range resource selection in free-ranging Impala in the Serengeti Ecosystem. Hourly model coefficients were regressed against single raised-cosine (noon-midnight activity) or double raised-cosine (dawn-dusk activity) daily pattern. Effect size (F statistic) and significance (P values) are given

Covariate	Noon / midnight		Dawn / dusk	
	F	*P*	F	*P*
Woody cover - outside	146.482	0.000	0.128	0.724
Shannon - outside	22.151	0.000	8.508	0.008
NDVI - outside	81.725	0.000	0.059	0.810
TRI - outside	49.870	0.000	0.034	0.854
Proximity water - outside	10.331	0.004	5.077	0.035
Woody cover - inside	16.569	0.001	10.281	0.004
Shannon - inside	79.285	0.000	1.119	0.302
NDVI - inside	6.786	0.016	4.986	0.036
TRI - inside	25.780	0.000	7.978	0.010
Proximity water - inside	78.459	0.000	1.703	0.205

## Discussion

Our study showed highest support for the risky places or ‘landscape of fear’ hypothesis in home-range placement and utilization of impalas inhabiting a ‘squeezed’ landscape [22, 76]. In multiple-predator systems, intra-guild competition and functionally different hunting tactics lead to spatial [77–79] and temporal [80–82] partitioning among carnivores which complicates balancing food acquisition and risk avoidance for their prey [83, 84]. The presence of humans is known to elicit a stronger behavioural [85, 86] and physiological [87, 88] response in ungulates compared to natural predators, whereby humans function as a ‘super-predator’ not only impacting herbivore space use, but also that of their predators [89]. Prey inhabiting such systems must therefore adjust their habitat selection in response to both carnivores and humans with contrasting risk patterns in space and time [76, 90]. The multi-scale resource selection shows that impalas, while inhabiting relatively confined home-ranges facilitated by their generalist mixed-feeding strategy, compensated for this limitation by spatially attuning their placement and selection of habitat to human disturbance and carnivore predation, and temporally fine-tuning their habitat utilization to balance foraging and safety according to circalunar and diel patterns. Impalas are a species of closed woodlands of fair forage quality in proximity to water. Inside SNP, where risk avoidance primarily revolves around predation, impalas selected stronger for more open heterogeneous vegetation but of poorer forage quality. Outside SNP, characterised by human disturbance and risk, as well as some degree of predation, impalas stayed farther from water sources and preferred to use more heterogeneous and rugged areas.

The greater selection for more closed woodland outside of SNP would indicate that impalas avoided spatial overlap with and sought visual cover from human disturbance [91], while the opposite was true when having to cope with carnivores alone [83]. Then, impalas attuned their nighttime habitat utilization to predator detection and fleeing opportunities, when they selected for open and flat terrain farther from water [48, 92, 93]. A similar avoidance of perceived risky places is a recurring response seen in ungulates, whereby avoidance of hunters [47, 83, 94, 95] and sit-and-pursue predators [47, 96, 97] is more pronounced compared to cursory predators. This effect in preference for open habitat is further strengthened during the full moon phase when carnivores have a higher nocturnal activity [81, 98]. Contrary to this, prey seek more closed and heterogeneous habitat during daytime that enhance forage quality [43, 99]; choosing more risky places when carnivores are less active [49, 96]. To secure forage quality, impalas placed their home-ranges in areas with higher NDVI, especially outside SNP where humans are present [91, 100]. While livestock and wildlife are spatio-temporally separated, and wildlife densities are generally lower, prey species may be able to seek out productive areas away from human activity [61]. However, inside SNP ungulate prey species have to share the available forage with the entire herbivore guild which may enhance forage competition and consequent niche partitioning, especially during the dry season [101, 102].

Mixed-feeding ungulates allow them to switch between grazing and browsing depending on forage quality [43]. This also (partially) releases impalas from the need to adjust their space use seasonally; instead, they showed diel patterns in their habitat utilization [91]. During dawn and dusk they showed a higher selection for covariates promoting forage opportunities: NDVI and Shannon vegetation diversity. Conversely, at midday these were slackened for a stronger selection for closed woodland to stay in cover from the heat [103]. The strong diel pattern in the selection for woody cover clearly indicates a spatio-temporal trade-off that balances diurnal thermoregulation and nocturnal predation risk [104]. Proximity to water plays a crucial role in balancing these aspects. While impalas drink when possible, their mixed feeding behaviour may better enable them to maintain water balance through forage intake [105]. This would reduce their need to be in close proximity to water and thereby allow them to avoid risky habitats at risky times [104]. The limited diel response to proximity to water may be due to the relative lower water dependence of impalas [105] limiting their exposure to predation [104]. The results on proximity to water mimic what has been found by others, with some nuances. Impalas in Kenya were found to stay closer to water sources in the reserve compared to the adjoining pastoral ranches, especially during drier years [106]. Similarly, in Hwange National Park, Zimbabwe, ungulates were observed to avoid water holes at night due to the increased risk of natural predation, while this diel pattern in their proximity to water was lacking in adjacent hunting areas, where instead the risk of diurnal hunting risk resulted in increased levels of nocturnal drinking [107]. They also found that ungulates were more vigilant and skittish around water holes in these hunting areas due to perceived predation risk [108]. However, to which extent predation risk mediates the interplay between food and water requirements and thermoregulation still needs to be better understood [104].

The impala is a gregarious species living in family or bachelor groups of varying herd size [42, 109]. In addition, mixed-species herds may occur [110, 111]. Aggregation enhances predator detection and dilutes the risk of being killed [112, 113], and thereby affect the time allocated to costly anti-predator behaviours [26, 42]. However, aggregation may also enhance foraging competition [110, 114]. Our analyses did not take the effect of herd size or social cohesion, cf [42], into account. Still, herd associations may well affect how impalas utilize their habitat; relaxing their selectivity for resources due to shared vigilance (lower individual risk may open up niche space). While herd size may reduce physiological stress in impalas, increased foraging behaviour may result in higher physiological stress, and ultimately fitness, especially when exposed to greater threats [87, 115, 116]. Forage quality has been shown to have a higher impact on stress in impalas than human disturbance, however, adjustments in risk allocation may well lead to spatially and/or temporal exclusion from foraging patches [87, 115].

Following the risk allocation hypothesis [26, 37, 38], the presence of a super-predator seems to have been of such an intensity and predictability that it changed the anti-predator responses in impala compared to the adjacent natural system with carnivores only. We found indication for a functional response in habitat selection in the human-altered landscapes for proximity to water. Impalas here reduced long-term spatial risk by avoiding water sources day and night. Within their home-ranges, short-term temporal risk however mimicked the diel patterns observed also within the protected area with natural predators only. Human activity in the form of agriculture, pastoralism or hunting/poaching have led to declines in ungulates [4] and their predators [117], as well as avoidance of human disturbance with a higher use of woodlands [5, 118, 119]. Vigilance and movement activity [28, 39, 120] in similar settings have however been shown to be mostly affected by short-term temporal risk of predation, more so in small browsers/mixed-feeding ungulates [29]. Although both predators and their prey have exhibited diel responses to the diurnal human activity pattern, this may also lead to increased nocturnal predation risk [121]. As long as predation risk or human disturbance is predictable, they are able to place their confined home-ranges in areas that best balance availability of forage and limited risk, and temporally ‘micro-manage’ their space use within. Still, some individuals did show marked seasonal and/or sudden changes in their home-range utilization. One individual in particular displayed a sudden relocation from LGCA 20 km westwards into SNP in circa one day. Eighteen days later she returned to the original site over a timespan of three days. As this individual’s home-range lay within a hunting concession, we suspect she was disturbed by hunters, cf [122]. If so, impalas are not entirely stuck in space as the title suggests but are able to adjust when risk exceeds certain thresholds.

## Conclusions

We tested risk allocation hypotheses in multi-scale resource selection for 36 GPS-tracked impalas in the Serengeti Ecosystem. Impalas compensated for the limitation of their confined home-ranges by spatially attuning their placement and selection of habitat based on predation risk inside SNP and both human disturbance and predation risk outside SNP and temporally fine-tuning their habitat utilization to balance foraging and exposure to the risk of predation according to circalunar and diel patterns.

## Data Availability

Supplementary data and R scripts to this article are available online on the Mendeley Data Repository at 10.17632/b8kt8y7ky3.2.

## Abbreviations

AIC: Akaike’s Information Criterion
GFCC: Global Forest Cover Change
GGR: Grumeti Game Reserve
GPS: Global Positioning System
IGR: Ikorongo Game Reserve
InOut: Locations with regard to the SNP boundaries
IWMA: IKONA Wildlife Management Area
LGCA: Loliondo Game Controlled Area
MCP: Minimum Convex Polygon
MGR: Maswa Game Reserve
Moon: Lunar cycle
NCA: Ngorogoro Conservation Area
NDVI: Normalized Difference Vegetation Index
ProxW: Proximity to water
RSF: Resource selection function
SNP: Serengeti National Park
SRTM: Shuttle Radar Topography Mission
SVD: Shannon vegetation diversity
TRI: Terrain ruggedness index
WC: Woody cover
